# Supplementary material for: Rad21 Haploinsufficiency Prevents ALT-Associated Phenotypes in Zebrafish Brain Tumors
Source: Genes (Basel). 2020 Nov 30;11(12):1442. doi: 10.3390/genes11121442 (PMC7760354; doi:10.3390/genes11121442)
Supplement: Supplementary file 1 [file genes-11-01442-s001.pdf]

A WB rad21

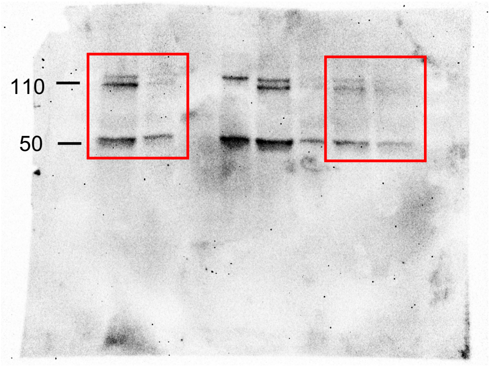

B CCircle dot blot

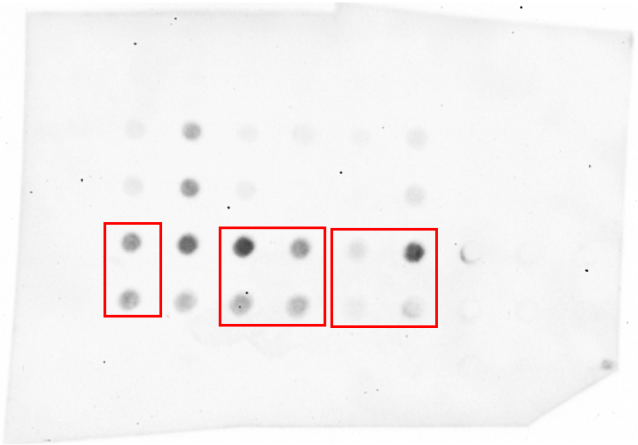

TERRA dot blot

C

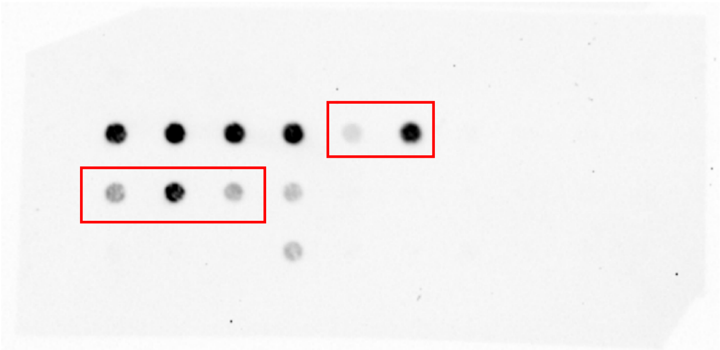

Supplementary figure 1.
Unedited dot blots used in figure 1D, 2H and 2L (boxed).
